# Triple Serosal Involvement in End-Stage Renal Disease (ESRD): A Case of Atypical Uremic Polyserositis With Elevated Serum-Ascites Albumin Gradient and Alkaline Phosphatase

**DOI:** 10.7759/cureus.89392

**Published:** 2025-08-05

**Authors:** Krishna Vamsy Polepalli, Samhitha Gonuguntla, Sadra Nokhostin, Anand Reddy

**Affiliations:** 1 Department of Internal Medicine, Rajarajeswari Medical College, Bangalore, IND; 2 Department of Internal Medicine, Texas Tech University Health Sciences Center, Odessa, USA; 3 Undergraduate Medical Education, St George's University of London, London, GBR; 4 Department of Nephrology, Texas Tech University Health Sciences Center, Odessa, USA

**Keywords:** end-stage renal disease (esrd), hemodialysis, high saag ascites, nephrogenic ascites, serosal effusions, uremic pericarditis, uremic pleuritis, uremic polyserositis

## Abstract

Uremic polyserositis is a rare and often underdiagnosed manifestation of end-stage renal disease (ESRD), typically characterized by concurrent or sequential inflammation of multiple serosal membranes. We report a diagnostically challenging case of a 40-year-old woman with ESRD on intermittent hemodialysis who presented with dyspnea following a missed dialysis session. Imaging revealed bilateral pleural effusions, a moderate-to-large pericardial effusion, and ascites. Fluid analyses from thoracentesis and pericardiocentesis showed sterile, hemorrhagic, and exudative effusions. Ascitic fluid demonstrated a serum-ascites albumin gradient (SAAG) of 2.8, an unusual finding, as uremic ascites typically presents with a SAAG of <1.1. Despite the elevated SAAG, liver imaging and function were unremarkable, and no signs of portal hypertension were observed. The patient improved clinically with dialysis and pericardial drainage but later decompensated after another missed session, with recurrence of effusions and similar biochemical features, which again resolved with renal replacement therapy.

This case highlights several atypical features of uremic polyserositis, including high SAAG ascites, markedly elevated alkaline phosphatase, and gamma-glutamyl transferase, in the absence of structural liver disease. These findings underscore how systemic inflammation and altered peritoneal dynamics in ESRD can produce biochemical patterns that mimic hepatic pathology. The strong temporal correlation between missed dialysis and symptom recurrence further supported a uremic etiology. Given the sterile nature of the effusions, lack of evidence for autoimmune or malignant processes, and consistent response to dialysis, uremic polyserositis was deemed the most plausible diagnosis.

In a clinical landscape where this entity is increasingly rare, our case emphasizes the importance of considering uremic polyserositis in patients with ESRD and recurrent, unexplained serosal effusions, especially when classical fluid parameters do not conform to expected patterns. Awareness of such atypical presentations is essential to avoid unnecessary interventions and to guide appropriate, dialysis-focused management.

## Introduction

Uremic polyserositis refers to the inflammation of multiple serosal membranes in the setting of advanced kidney failure and uremia. While isolated involvement of the pericardium, pleura, or peritoneum is relatively common in end-stage renal disease (ESRD), simultaneous or sequential involvement of all three serosal compartments is rare and underrecognized in modern nephrology practice. The underlying mechanism involves the accumulation of uremic toxins, systemic inflammation, and capillary leakage, leading to sterile and exudative effusions across serosal surfaces [1-3].

In the pre-dialysis era, polyserositis was frequently encountered due to delayed recognition of uremia; however, its incidence has declined significantly with routine and early initiation of renal replacement therapy. Nevertheless, non-compliance with dialysis or suboptimal delivery continues to pose risks for the development of this condition [4].

A diagnostic challenge arises when the fluid analysis deviates from classical patterns, as seen in our case. For example, uremic ascites is expected to have a serum-ascites albumin gradient (SAAG) of <1.1, yet our patient presented with a SAAG of >2.8, typically indicative of portal hypertension. Although volume overload due to missed hemodialysis sessions can result in serosal effusions, these are typically transudative and non-hemorrhagic. In contrast, uremic effusions tend to be exudative, serosanguinous, and lymphocyte-predominant, suggesting an underlying inflammatory etiology, such as uremic polyserositis, rather than hydrostatic imbalance alone. This discordance necessitates careful integration of imaging, clinical context, and response to therapy. Similarly, the simultaneous presence of hemorrhagic pleural and pericardial effusions raised the differential of infection, malignancy, or autoimmune disease, all of which were ultimately ruled out.

In this report, we present a unique case of uremic polyserositis with high SAAG ascites, recurrent pleuropericardial effusions, and full biochemical resolution following dialysis. We highlight how atypical presentations can mimic other serious conditions and emphasize the importance of diagnostic caution and therapeutic alignment with uremic management.

## Case presentation

A 40-year-old African American woman with a history of insulin-dependent type 2 diabetes mellitus, hypertension, and ESRD secondary to diabetic and hypertensive nephrosclerosis presented with progressive shortness of breath following a missed hemodialysis session. She had been undergoing thrice-weekly hemodialysis (Tuesday, Thursday, and Saturday) for the past 10 months and was listed for renal transplantation.

Her past medical history included systemic lupus erythematosus (SLE), depression, and prior substance use (marijuana, methamphetamine, and cocaine); she had been abstinent for the past 10 months. Surgical history was notable for appendectomy and cholecystectomy.

On presentation, her oxygen saturation on room air was 70%, and she appeared dyspneic but hemodynamically stable. Laboratory investigations are summarized in Table 1. Troponin was mildly elevated, consistent with type 2 non-ST elevation myocardial infarction in the setting of demand ischemia. Echocardiography revealed mildly increased left ventricular wall thickness, preserved chamber size, an ejection fraction of 50%-55%, mild right ventricular systolic dysfunction, and a moderate-to-large pericardial effusion. An electrocardiogram showed no acute changes. Coronary angiography performed four months earlier during transplant evaluation demonstrated normal coronary anatomy.

**Table 1:** Admission laboratory parameters

Parameter	Value	Reference range
White blood cell count	6.8×10³/µL	4.5–11×10³/µL
Neutrophils	73%	40%–70%
Red blood cell count	2.41×10⁶/µL	4.2–5.4×10⁶/µL (female)
Hemoglobin	6.6 g/dL	12–16 g/dL (female)
Mean corpuscular volume	83.4 fL	80–100 fL
Platelets	319×10³/µL	150–450×10³/µL
Sodium	130 mmol/L	135–145 mmol/L
Potassium	4.3 mmol/L	3.5–5.0 mmol/L
Chloride	88 mmol/L	98–108 mmol/L
Calcium	10.4 mg/dL	8.6–10.0 mg/dL
Aspartate aminotransferase	32 U/L	10–40 U/L
Alanine aminotransferase	40 U/L	10–40 U/L
Total bilirubin	7.9 mg/dL	0.1–1.2 mg/dL
Alkaline phosphatase	1516 U/L	30–300 U/L
Gamma-glutamyl transferase	950 U/L	0–51 U/L
Anion gap	15	10–14
Prothrombin time	17.8 seconds	11–13.5 seconds
Creatinine	3.4 mg/dL	0.7–1.2 mg/dL
Blood urea nitrogen	32 mg/dL	6–20 mg/dL

**Figure 1:**
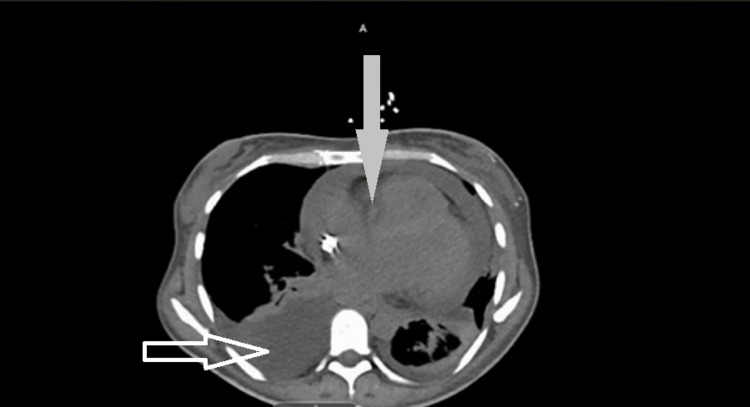
CT of the chest without contrast (axial view) demonstrating a moderate pericardial effusion (solid white arrow) and a left-sided pleural effusion (transparent white arrow)

Initial imaging performed at a small county hospital, where the patient first presented, included a CT thorax, which demonstrated bilateral pleural effusions (right greater than left) and a moderate-to-large pericardial effusion. These findings are visually depicted in Figure 1, highlighting both the pleural and pericardial effusions. Based on these findings and her respiratory distress, the patient was empirically started on doxycycline and ceftriaxone for presumed infectious pleuritis in accordance with local guidelines. However, due to persistent symptoms and diagnostic uncertainty, she was transferred to our facility for higher-level care and further evaluation.

The patient had experienced multiple prior episodes of serosal effusions requiring drainage. She underwent bilateral thoracentesis, which yielded 450 mL from the right and 325 mL from the left pleural spaces. A therapeutic paracentesis drained 250 mL of ascitic fluid.

A diagnostic thoracentesis was performed on the day of admission, draining 850 mL of hemorrhagic pleural fluid. Pleural fluid analysis findings are summarized in Table 2. Light’s criteria confirmed the effusion to be exudative. Following thoracentesis, the patient was restarted on hemodialysis. After her first dialysis session, creatinine improved to 2.3 mg/dL and blood urea nitrogen (BUN) to 18 mg/dL, along with symptomatic relief.

**Table 2:** Pleural fluid analysis

Parameter	Result	Reference range
Appearance	Hemorrhagic	Clear, straw-colored
Volume	850 mL	<50 mL (physiological)
Glucose	180 mg/dL	~ Equal to serum glucose
Lactate dehydrogenase	78 U/L	<200 U/L
Protein	4.4 g/dL	<3.0 g/dL (transudate)
Serum protein	8.7 g/dL	6.0–8.3 g/dL
Serum albumin	4.9 g/dL	3.5–5.0 g/dL
Light’s criteria	Exudative	

Given her known history of SLE, autoimmune serositis was considered early in the diagnostic workup. However, complement levels were within or above normal limits--C3 was 178 mg/dL (reference range: 90-180 mg/dL) and C4 was 46 mg/dL (reference range: 10-40 mg/dL). Autoantibody screening, including anti-dsDNA, SSA, SSB, Smith, and RNP antibodies, was negative. These findings, along with the absence of clinical signs of lupus flare such as rash, arthritis, or cytopenia, made an autoimmune etiology unlikely.

Subsequently, a CT scan of the abdomen and pelvis was performed, which showed new diffuse liver heterogeneity, leading to a new diagnosis of liver cirrhosis, presumed to be secondary to metabolic dysfunction. Liver enzymes were within normal limits, with an aspartate aminotransferase of 38 U/L and alanine aminotransferase of 40 U/L, and there was no radiographic evidence of portal hypertension at that time.

On day 3 of admission, a subxiphoid pericardial window was performed due to persistent effusion, draining 115 mL of bloody, turbid fluid. Pericardial fluid characteristics are summarized in Table 3. Over the subsequent days, alkaline phosphatase (ALP) levels initially rose, peaking at 1892 U/L on day 6, then gradually declined with dialysis and fluid drainage.

**Table 3:** Pericardial fluid analysis

Parameter	Result	Reference range
Appearance	Bloody, turbid	Clear, straw-colored
Volume	115 mL	Usually <50 mL (physiological); >50 mL suggests pathological effusion
Red blood cell count	481,640/µL	<5,000/µL; >100,000/µL may indicate hemorrhagic effusion
White blood cell count	7,140/µL	<1,000/µL
Differential	Predominantly macrophages and lymphocytes	Mixed; varies by etiology
Cytology	Negative for malignancy	No malignant cells

**Figure 2:**
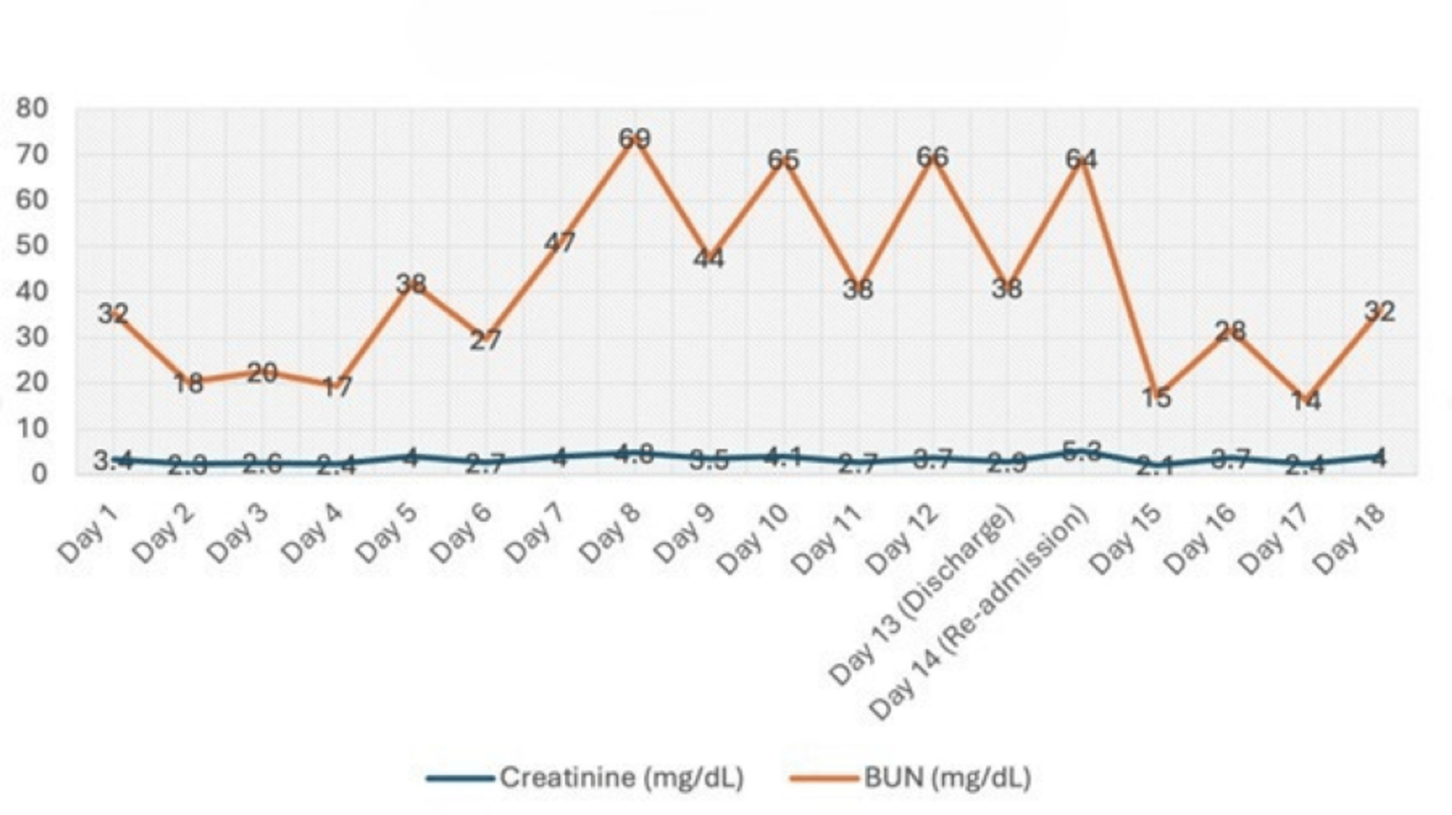
Creatinine and blood urea nitrogen trends

In parallel, serum creatinine and BUN levels closely reflected dialysis adherence, with marked improvements following hemodialysis and deterioration after missed sessions. This trend is summarized in Figure 2.

The patient was discharged after 13 days of regular hemodialysis sessions and did not require any repeat drainage of serosal effusions during this period. At that time, creatinine was 2.9 mg/dL and BUN was 38 mg/dL, reflecting biochemical improvement consistent with dialysis.

**Figure 3:**
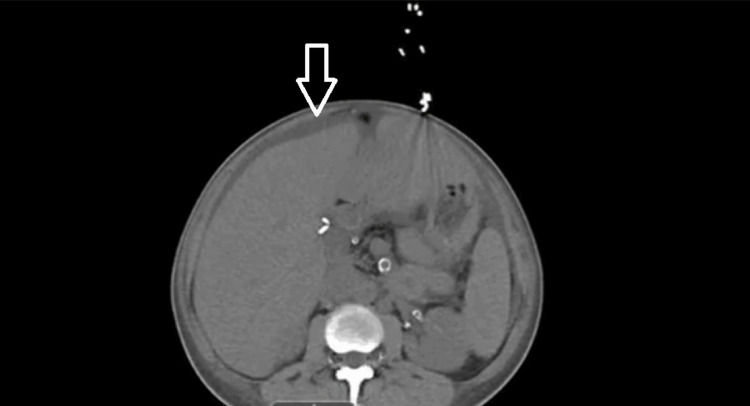
CT of the abdomen and pelvis without contrast (axial view) showing ascites, indicated by the transparent white arrow along the perihepatic region

However, she was readmitted within 4 days following another missed dialysis session, this time presenting with left lower quadrant abdominal pain radiating to the back. Repeat imaging revealed mild-to-moderate ascites, left lower lobe atelectasis or pneumonia, and bladder wall thickening suggestive of cystitis. Figure 3 demonstrates the presence of ascites as seen on repeat imaging. A diagnostic paracentesis was performed, yielding 30 mL of dark yellow ascitic fluid. Fluid characteristics are summarized in Table 4. The findings were not suggestive of spontaneous bacterial peritonitis or malignancy.

**Table 4:** Ascitic fluid analysis

Parameter	Result	Reference range
Appearance	Dark yellow	Clear, pale yellow
Volume	30 mL	Variable; usually <50 mL (physiological)
White blood cell count	135/µL	<250/µL
Red blood cell count	1131/µL	<1,000/µL (typically normal); >10,000/µL = hemorrhagic ascites
Cell differential	Predominantly macrophages and lymphocytes	Predominantly mononuclear cells
Lactate dehydrogenase	100 U/L	<225 U/L
Ascitic albumin	2.1 g/dL	—
Serum albumin	4.9 g/dL	3.5–5.0 g/dL
Serum-ascites albumin gradient	2.8	>1.1 = portal hypertension; <1.1 = non-portal causes
Culture and cytology	Sterile; benign	Sterile, no malignant cells

At the time of readmission, creatinine had risen to 5.4 mg/dL and BUN to 64 mg/dL, consistent with recurrent uremia. ALP levels also began rising again, increasing from 1228 U/L on the day of re-admission, despite no identifiable new source of infection, biliary obstruction, or malignancy on follow-up imaging and nuclear liver-spleen scan.

Given the recurrent exudative effusions involving pleural, pericardial, and peritoneal compartments, negative infectious, autoimmune, and neoplastic workup, and a consistent temporal association with missed dialysis sessions, a diagnosis of uremic polyserositis was established. The patient’s clinical improvement with dialysis and serous fluid drainage further supported this diagnosis.

## Discussion

Uremic polyserositis is an uncommon but clinically significant complication of ESRD, characterized by inflammation of multiple serous membranes, including the pericardium, pleura, and peritoneum. Though historically more prevalent in the pre-dialysis era, its incidence has declined with the routine implementation of renal replacement therapy. However, it can still occur in patients with delayed diagnosis, dialysis non-adherence, or inadequate dialysis dosing, as seen in our patient.

The pathogenesis is multifactorial and primarily attributed to the accumulation of uremic toxins and systemic inflammation, which increase capillary permeability and lead to sterile, often hemorrhagic, exudative effusions. This process may involve one or multiple serosal surfaces simultaneously or sequentially. Although the precise mechanisms remain unclear, studies have implicated cytokine elevation, complement activation, and impaired lymphatic drainage [2-5].

In this case, all three serosal compartments were affected. The patient developed a pericardial effusion requiring surgical drainage, hemorrhagic pleural effusions, and small-volume ascites. These events were temporally associated with missed dialysis sessions and improved consistently with intensified hemodialysis and fluid drainage, supporting the diagnosis of uremic polyserositis.

While the patient’s missed dialysis sessions may have contributed to fluid accumulation, several features argue against simple volume overload as the primary cause. The pleural and pericardial effusions were hemorrhagic and exudative, with lymphocyte-predominant cytology hallmarks of uremic serositis rather than transudative effusions caused by hydrostatic pressure [6]. In volume overload, effusions are usually clear, straw-colored, and transudative in nature. Additionally, the resolution of symptoms following dialysis and drainage further supports the inflammatory nature of the effusions.

Although the patient had a known history of SLE, her clinical presentation and response to treatment were not consistent with an autoimmune flare. The absence of rash, cytopenia, or renal involvement, combined with negative autoantibody testing and normal complement levels, made lupus serositis unlikely. The rapid resolution of symptoms with dialysis alone, without immunosuppressive therapy, further supported a uremic etiology.

The pleural fluid in our patient met Light’s criteria for an exudate, with elevated protein and sterile characteristics, consistent with uremic pleuritis [6]. Pericardial involvement, likewise, manifested as a hemorrhagic effusion requiring a subxiphoid pericardial window. Surgical drainage is indicated when tamponade physiology or persistent hemodynamic compromise occurs despite dialysis [7].

A unique diagnostic complexity was presented by the ascitic fluid findings. The patient had a SAAG of 2.8, classically indicative of portal hypertension. However, several features argued against this diagnosis: (1) imaging, including CT and magnetic resonance cholangiopancreatography (MRCP), showed no evidence of cirrhosis, portal vein thrombosis, or hepatic congestion; (2) liver enzymes (aspartate aminotransferase: 38 U/L and alanine aminotransferase: 40 U/L) were within normal limits; international normalized ratio (INR) was also normal, indicating preserved synthetic function; and (3) the ascitic fluid was low in volume, non-progressive, sterile, and showed a predominance of macrophages and lymphocytes. Although uremic ascites typically presents with a SAAG of <1.1, studies have shown that a high SAAG may still occur in patients with ESRD, altered peritoneal dynamics, and high serum albumin [8]. Therefore, a high SAAG alone should not exclude uremic polyserositis, particularly in the absence of radiologic or clinical evidence of portal hypertension.

Another diagnostic challenge in this case was the markedly elevated ALP, accompanied by a significantly elevated gamma-glutamyl transferase, suggestive of a hepatobiliary origin rather than a bone source. However, imaging, including abdominal CT and MRCP, revealed no biliary obstruction, cirrhosis, or liver mass. Transaminases and bilirubin were normal or mildly elevated, and INR remained within normal range, arguing against intrinsic hepatocellular injury. One plausible explanation is functional cholestasis or inflammation-driven hepatic enzyme derangement, a phenomenon reported in patients with ESRD, where systemic inflammation and uremic toxins may alter hepatic enzyme expression without structural liver disease [9,10]. This is further supported by the temporal correlation of ALP fluctuations with missed dialysis sessions and clinical deterioration.

The cornerstone of management for uremic polyserositis is prompt and adequate dialysis. In most cases, intensified hemodialysis alone is sufficient to resolve effusions. However, large or symptomatic effusions, especially those causing tamponade or respiratory compromise, may necessitate procedural drainage, as in our patient. While empiric antibiotics are often initiated by pending cultures, effusions in uremic serositis are usually sterile. Corticosteroids are not routinely recommended unless an autoimmune etiology is suspected. Close monitoring is essential in patients with known non-compliance or other risk factors for recurrence [11].

Although uremic pericarditis and pleuritis are more frequently reported, simultaneous involvement of the pericardium, pleura, and peritoneum is rare and may be underrecognized. This case underscores the importance of correlating clinical timing, fluid analysis, and response to dialysis to avoid unnecessary interventions for presumed infection or malignancy.

Teaching points

Uremic polyserositis can manifest with atypical biochemical profiles, including high SAAG ascites, which may lead to diagnostic uncertainty. The diagnosis is primarily one of exclusion, supported by a temporal relationship with missed dialysis sessions and the presence of sterile inflammatory fluid. Despite the variability in laboratory findings, clinical improvement following dialysis remains the most consistent diagnostic and therapeutic indicator. It is essential for clinicians to recognize these atypical presentations in patients with ESRD and to maintain a high index of suspicion when evaluating serosal effusions in this population.

## Conclusions

This case underscores the importance of maintaining a high index of suspicion for uremic polyserositis in patients with ESRD, especially when multiple serosal compartments, pericardial, pleural, and peritoneal, are involved simultaneously. The temporal association between symptom onset and missed or delayed dialysis sessions remains a key diagnostic clue. Although fluid analysis may reveal atypical findings such as high SAAG ascites or hemorrhagic pleural effusion, the absence of hepatic disease, sterile inflammatory profile, and significant improvement with intensified dialysis should prompt consideration of a uremic etiology.

While surgical drainage may be necessary in cases of hemodynamic compromise, most effusions resolve with optimized medical management. Early recognition and treatment can reduce morbidity, prevent unnecessary invasive procedures, and optimize resource utilization. This case highlights a diagnostic challenge in nephrology and reinforces the need to revisit the conventional understanding of serosal fluid characteristics in ESRD. We advocate for further studies to characterize biochemical variants of uremic effusions, which may improve early identification and targeted treatment of this underrecognized condition.
